# The Role of Nrf2 Signaling in PPAR*β*/*δ*-Mediated Vascular Protection against Hyperglycemia-Induced Oxidative Stress

**DOI:** 10.1155/2018/5852706

**Published:** 2018-06-25

**Authors:** Rosario Jimenez, Marta Toral, Manuel Gómez-Guzmán, Miguel Romero, Manuel Sanchez, Ayman M. Mahmoud, Juan Duarte

**Affiliations:** ^1^Department of Pharmacology, School of Pharmacy, University of Granada, Granada, Spain; ^2^Instituto de Investigación Biosanitaria de Granada (ibs.GRANADA), Granada, Spain; ^3^Ciber-Enfermedades Cardiovasculares (CIBERCV), Granada, Spain; ^4^Physiology Division, Department of Zoology, Faculty of Science, Beni-Suef University, Beni Suef, Egypt; ^5^Department of Endocrinology, Diabetes and Nutrition, Charité-University Medicine Berlin, Berlin, Germany; ^6^Department of Endocrinology, Diabetes and Nutrition at the Center for Cardiovascular Research (CCR), Charité-University Medicine Berlin, Berlin, Germany

## Abstract

Hyperglycemia induces oxidative stress and plays a substantial role in the progression of vascular diseases. Here, we demonstrated the potentiality of peroxisome proliferator-activated receptor (PPAR)*β*/*δ* activation in attenuating high glucose-induced oxidative stress in endothelial cells and diabetic rats, pointing to the involvement of nuclear factor erythroid 2-related factor 2 (Nrf2). HUVECs exposed to high glucose showed increased levels of reactive oxygen species (ROS) and upregulated NOX-2, NOX-4, Nrf2, and NQO-1 effects that were significantly reversed by the PPAR*β*/*δ* agonists GW0742 and L165041. Both PPAR*β*/*δ* agonists, in a concentration-dependent manner, induced transcriptional and protein upregulation of heme oxygenase-1 (HO-1) under low- and high-glucose conditions. All effects of PPAR*β*/*δ* agonists were reversed by either pharmacological inhibition or siRNA-based downregulation of PPAR*β*/*δ*. These *in vitro* findings were confirmed in diabetic rats treated with GW0742. In conclusion, PPAR*β*/*δ* activation confers vascular protection against hyperglycemia-induced oxidative stress by suppressing NOX-2 and NOX-4 expression plus a direct induction of HO-1; with the subsequent downregulation of the Nrf2 pathway. Thus, PPAR*β*/*δ* activation could be of interest to prevent the progression of diabetic vascular complications.

## 1. Introduction

Uncontrolled hyperglycemia in diabetes is linked to many micro- and macrovascular complications [1]. Several lines of evidence advocate the role of endothelial dysfunction in the development of cardiovascular (CV) disease [2]. Endothelial dysfunction (ED) represents the key early step and the prognostic marker of diabetes-associated vascular complications and is characterized by diminished bioavailability of vasodilators [3]. In hyperglycemia, oxidative stress and elevated levels of reactive oxygen species (ROS) in the vessels are strongly linked to ED [4]. Overproduction of ROS has been reported to result in a wide account of potentially damaging intermediates that damage DNA, proteins, membrane structure, and metabolic activity, thereby causing cellular dysfunction and cell death, which lastly lead to alterations in the balance between prooxidants and antioxidant arising several diseases as an outcome [5].

The nuclear factor erythroid 2-related factor 2 (Nrf2) is a basic leucine zipper protein that suppresses oxidative stress through activating the transcription of multiple defensive and antioxidant genes [6]. In the endothelium, Nrf2 has been reported to be activated via increased ROS generation [7] and multiple studies have demonstrated the effectiveness of Nrf2 signaling in counteracting the deleterious repercussion of ROS in the endothelium [8, 9].

Peroxisome proliferator-activated receptor-*β*/*δ* (PPAR*β*/*δ*) is a member of a group of nuclear receptors that play diverse roles in metabolism, development, and cellular differentiation. PPAR*β*/*δ* regulates numerous genes implicated in glucose homeostasis, and fatty acid metabolism is therefore ubiquitously expressed in metabolically active tissues [10, 11]. In high-fat diet- (HFD-) induced type 2 diabetes, PPAR*β*/*δ* activation improves glucose and lipid metabolism and confers vascular protection [12]. Previous studies have demonstrated that, independent of their metabolic actions, PPAR*β*/*δ* agonists improved endothelial dysfunction in animal models of diseases associated with increased ROS, such as obesity, diabetes, and hypertension [12–16]. In addition, activation of PPAR*β*/*δ* reestablished the altered insulin signaling pathway in human endothelial cells exposed to high glucose levels [17] and improved vascular reactivity in the arteries of diabetic rodents [13, 14, 18]. Theses endothelium protective effects seem to be mediated via inhibition of mitochondrial- [17] and nicotinamide adenine dinucleotide phosphate (NADPH) oxidase-derived ROS production [14] and ERK1/2 activation [17]. Although PPAR*β*/*δ* activation protects the endothelium against diabetes-associated oxidative damage by diminishing the sources of ROS in the vasculature, nothing has yet been reported on the role of Nrf2 signaling in mediating the protective effect of PPAR*β*/*δ*. Therefore, we demonstrated the modulatory effect of PPAR*β*/*δ* activation on Nrf2 and its target genes using *in vitro* high glucose-induced endothelial cell model and *in vivo* diabetic animal model.

## 2. Materials and Methods

### 2.1. Cell Culture and Treatments

Human umbilical vein endothelial cells (HUVECs), isolated from cord veins as previously reported [14] with some adaptations, were used in all *in vitro* experiments. The isolated cells were cultured in medium 199 (M199), and cells from passage 2–5 were used for the experiments. Following a 2 h serum starvation, HUVECs were treated with 10^−7^–10^−6^ M of either GW0742 or L165041 for 24 h in low-glucose (LG; 5 mM) or high-glucose (HG; 30 mM) condition. Other HUVECs were preincubated with 10^−6^ M GSK0660, PPAR*β*/*δ* antagonist, for 1 h before treatment with the PPAR*β*/*δ* agonists.

### 2.2. Transfection of PPAR*β*/*δ* siRNA

Confluent HUVECs were transfected with PPAR*β*/*δ* or control siRNAs (Dharmacon, Lafayette, CO, USA) using Lipofectamine RNAiMAX (Invitrogen, Carlsbad, CA, USA) for 48 h [19]. The efficiency of PPAR*β*/*δ* siRNAs transfection was affirmed using qPCR and Western blotting.

### 2.3. Assay of Intracellular ROS

HUVECs were seeded in 96-well plates and treated with PPAR*β*/*δ* agonists and/or antagonist in LG or HG M199 and then incubated with 5 *μ*M 2′-7′-dichlorodihydrofluorescein diacetate (CM-H_2_DCFDA) at 37°C for 30 min. After washing, the fluorescence intensity was determined using a microplate reader (Fluorostart, BMG Lab Technologies, Offenburg, Germany).

### 2.4. Gene Expression Analysis

The effect of PPAR*β*/*δ* activation on the expression of Nrf2, NAD(P)H quinone dehydrogenase 1 (NQO-1), heme oxygenase-1 (HO-1), NOX-4, NOX-2, and NOX-1 was evaluated using qPCR. Briefly, total RNA was isolated, quantified, and reverse transcribed into cDNA. qPCR was performed as we previously reported [14], using the primers set described in Table 1. The obtained data were analyzed using the 2^−∆∆Ct^ method with *β*-actin or GAPDH as housekeeping genes and normalized to the control group.

### 2.5. Western Blot Analysis

Proteins from HUVECs were separated on SDS-PAGE and transblotted onto PVDF membranes [14, 15]. The blots were probed with antibodies (Santa Cruz Biotechnology, CA, USA) against Nrf2, NQO-1, HO-1, and *α*-actin (Santa Cruz Biotechnology, CA, USA) followed by the secondary antibody. The ECL system (Amersham, UK) was used to develop and visualize the blots which were then analysed using Scion Image-Release Beta 4.02 software (http://scion-image.software.informer.com/).

### 2.6. Animal Experiments

The effect of PPAR*β*/*δ* activation on Nrf2 signaling in the aorta was investigated using male Wistar rats weighing 280–320 g and maintained on a 12 h light/dark cycle at 24 ± 1°C with standard rat chow water ad libitum. The experimental protocol was approved by the institutional review board of the University of Granada (Spain), and all procedures were conducted according to the guidelines for the Care and Use of Laboratory Animals published by National Institutes of Health. Type 1 diabetes was induced by injection 50 mg^.^kg^−1^ streptozotocin (STZ) (Sigma-Aldrich) [14] dissolved in a citrate buffer (pH 4.5) into the tail vein. Three days after intravenous (i.v.) STZ injection, animals faster for 18 h were screened using an Accu-Chek Aviva glucometer (Roche Diagnostics S.L., Barcelona, Spain) and rats with blood glucose 200 mg/dL^−1^ or above were selected. In parallel, control rats received a single i.v. of citrate buffer. The control and diabetic animals were divided into 4 groups (*N* = 8–10) as follows:

Group I (control): rats received the vehicle 1% methylcellulose by gavage for 5 weeks.

Group II (GW-treated): rats received 5 mg/kg/day GW0742 dissolved in 1% methylcellulose [20, 21] by gavage daily for 5 weeks.

Group III (diabetic): diabetic rats received the vehicle 1% methylcellulose by gavage for 5 weeks.

Group IV (GW-treated diabetic): diabetic rats received 5 mg/kg/day GW0742 dissolved in 1% methylcellulose [20, 21] by gavage.

At the end of treatments, the rats were sacrificed and dissected and the thoracic aortas were removed. Parts of the aorta were cut into rings which were cryopreserved in 0.1 M PBS plus 30% sucrose for 1 h, included in OCT medium and kept frozen −80°C, while other rings were used to assay NADPH oxidase activity. Other samples of the thoracic aorta were used to isolate RNA, and the gene expression was determined as described above.

### 2.7. In Situ Detection of Vascular Superoxide Anion Production

The frozen aortic rings were cut into 10 *μ*m cross sections by using a cryostat (Microm International Model HM 500 OM). The sections were stained with 10 *μ*M dihydroethidium (DHE) for 30 min in the dark at room temperature followed by counterstaining with DAPI. In the following 24 h, the DHE/DAPI-stained sections were examined using a fluorescence microscope (Leica DM IRB, Wetzlar, Germany) and images were captured. The DHE and DAPI fluorescence was quantified using ImageJ (version 1.32j, http://imagej-1-32j.updatestar.com/). The relative level of superoxide was estimated from the DHE/DAPI fluorescence ratio [22]. The specificity of the assay was tested using the superoxide scavenger tiron.

### 2.8. Assay of NADPH Oxidase Activity

The activity of NADPH oxidase in thoracic aortic rings of control and diabetic rats was assayed by the lucigenin-enhanced chemiluminescence assay as previously described [14]. Briefly, aortic rings were incubated in HEPES-buffered solution (pH 7.4) to which 100 *μ*M NADPH was added. Five *μ*M lucigenin was added, and the luminescence was recorded at 5 sec intervals over a 200 sec using in a luminometer (Lumat LB 9507, Berthold, Germany). After subtracting the basal values, the relative luminescence units (RLU)/min/mg dry tissue was used to express NADPH oxidase activity.

### 2.9. Statistical Analysis

All data were analyzed using GraphPad Prism 5 (GraphPad Software, San Diego, CA, USA). The results were expressed as mean ± SEM, and comparisons were made using Student's *t*-test or one-way ANOVA followed by Bonferroni's post hoc analysis. A *P* value < 0.05 was considered statistically significant.

## 3. Results

### 3.1. High Glucose Increases Nrf2 and Its Target Genes in HUVECs

**Figure 1:**
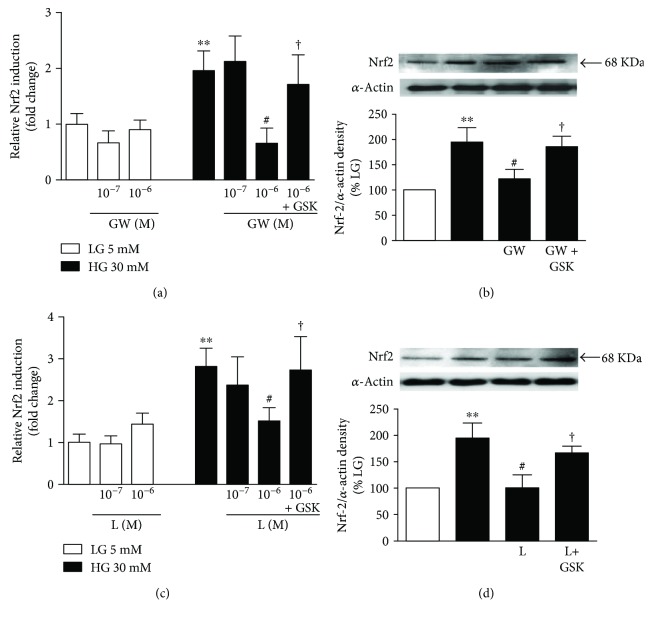
Effects of PPAR*β*/*δ* agonists on Nrf2 expression. (a, c) mRNA and (b, d) protein expression of Nrf2 in HUVECs exposed to low (5 mM, LG) or high glucose (30 mM, HG) for 24 h with or without GW0742 (GW) or L165041 (L) alone or preincubated with the PPAR*β*/*δ* antagonist GSK0660 (GSK). mRNA data presented as a ratio of arbitrary units of mRNA (2^−ΔΔCt^). All data are mean ± SEM (*n* = 8), and experiments were repeated at least three times independently. Protein data presented as densitometric values and protein band normalized to the corresponding *α*-actin; the bands are representative of *n* = 3–5. ^∗∗^*P* < 0.01 versus LG. ^#^*P* < 0.05 versus HG. †*P* < 0.05 versus L and GW column, respectively.

**Figure 2:**
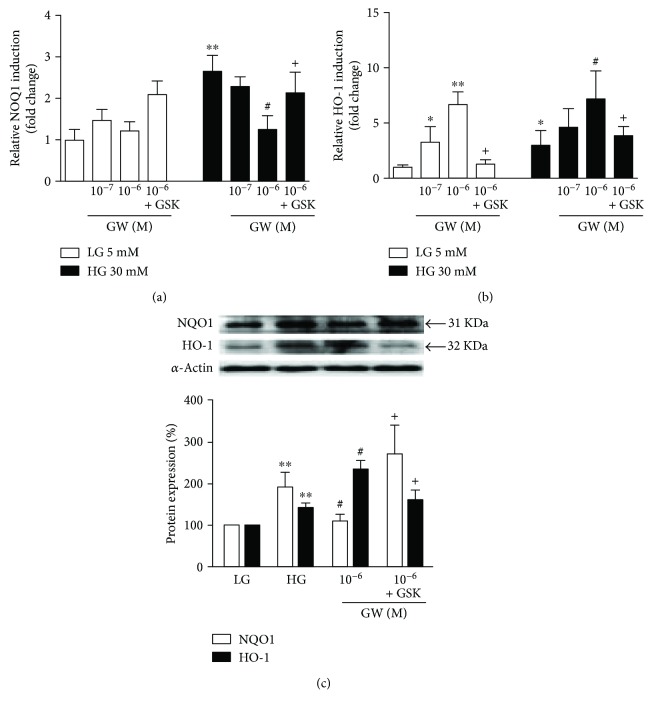
Effect of PPAR*β*/*δ* agonist, GW0742, on Nrf2 target gene induction. (a, b) mRNA and (c) protein expression of NOQ-1 and HO-1. HUVECs, exposed to low- (LG) or high-glucose (HG) medium for 24 h, were coincubated with GW0742 (GW) alone or preincubated with the GSK0660 (GSK) followed by GW0742. mRNA data presented as a ratio of arbitrary units of mRNA (2^−ΔΔCt^). All data are mean ± SEM (*n* = 8), and experiments were repeated at least three times independently. Protein data presented as densitometric values and protein band normalized to the corresponding *α*-actin; the bands are representative of *n* = 3–5. ^∗^*P* < 0.05 and ^∗∗^*P* < 0.01 versus LG. ^#^*P* < 0.05 versus HG. ^+^*P* < 0.05 versus GW column.

**Figure 3:**
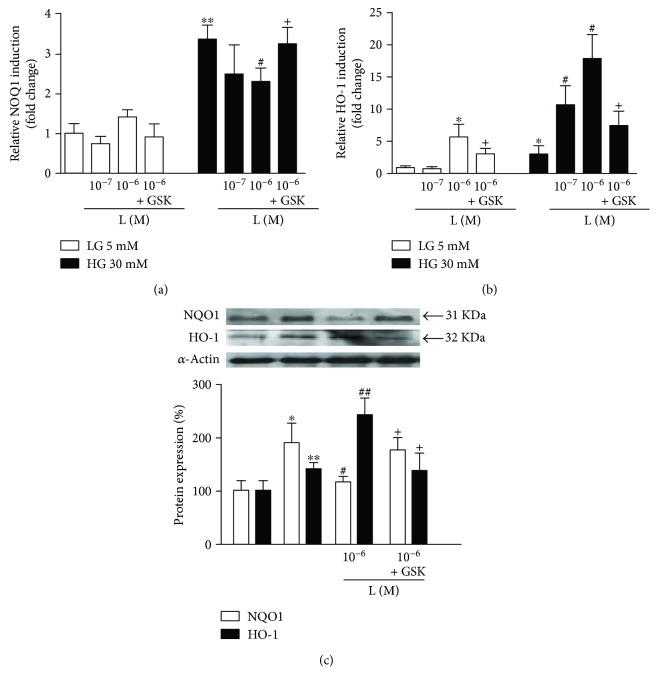
Effect of PPAR*β*/*δ* agonist, L165041, on Nrf2 target genes. (a, b) mRNA and (c) protein expression of NOQ-1 and HO-1. HUVECs, exposed to low- (LG) or high-glucose (HG) medium for 24 h, were coincubated with L165041 (L) alone or preincubated with the GSK0660 (GSK) followed by L165041. mRNA data presented as a ratio of arbitrary units of mRNA (2^−ΔΔCt^). All data are mean ± SEM (*n* = 8), and experiments were repeated at least three times independently. Protein data presented as densitometric values and protein band normalized to the corresponding *α*-actin; the bands are representative of *n* = 3–5. ^∗^*P* < 0.05 and ^∗∗^*P* < 0.01 versus LG. ^#^*P* < 0.05 and ^##^*P* < 0.01 versus HG. ^+^*P* < 0.05 versus L column.

HUVECs were incubated in HG medium for 24 h, and the expression of Nrf2, NQO-1, and HO-1 was assayed. HG induced a significant increase in the expression of Nrf2 both mRNA and protein (Figure 1). Similarly, the expression of NQO-1 and HO-1 gene as well as protein was significantly increased in HUVECs exposed to HG medium as compared to the LG one as represented in Figures 2 and 3, respectively.

### 3.2. Effects of PPAR*β*/*δ* Agonists on High Glucose-Induced Changes in Expression of Nrf2 and Its Target Genes

HUVECs treated with 10^−7^ and 10^−6^ M GW0742 (Figure 1(a)) or L165041 (Figure 1(c)) for 24 h in LG medium showed nonsignificant changes in the expression levels of Nrf2. On the contrary, coincubation with PPAR*β*/*δ* agonists downregulated Nrf2 mRNA (Figures 1(a) and 1(c)) and protein expression levels (Figures 1(b) and 1(d)) in HG-induced HUVECs. Coincubation of the HUVECs with 10^−6^ M of the PPAR*β*/*δ* antagonist GSK0660 inhibited the effects exerted by PPAR*β*/*δ* agonists (Figure 1).

The PPAR*β*/*δ* agonists did not alter the expression of NQO-1 mRNA and protein in normal experimental conditions. However, in HUVECs incubated in high-glucose medium, NQO-1 mRNA and protein levels were decreased by either GW0742 (Figures 2(a) and 2(c)) or L165041 (Figures 3(a) and 3(c)). Coincubation with GSK0660 markedly abolished the effect of PPAR*β*/*δ* agonists on NQO-1 expression, involving PPAR*β*/*δ* activation.

In contrast, in both conditions, the incubation with GW0742 (Figures 2(b) and 2(c)) and L165041 (Figures 3(b) and 3(c)) upregulated the expression of HO-1 an effect that was abolished by coincubation with GSK0660.

**Figure 4:**
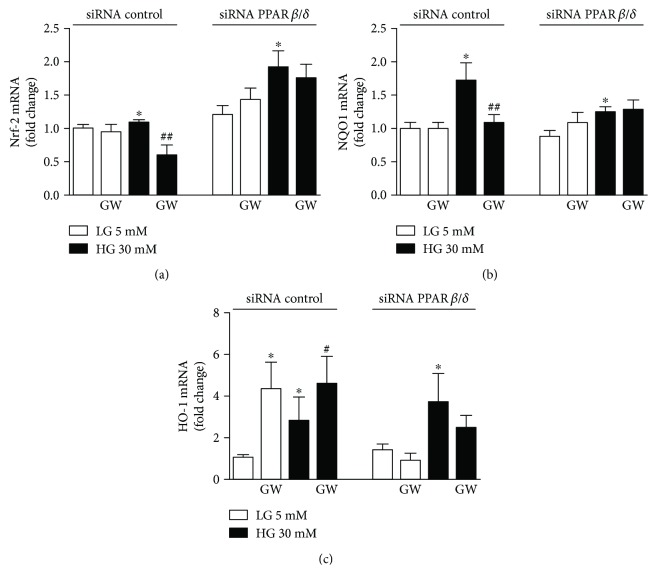
Role of the PPAR*β*/*δ* activation on the Nrf2/ARE pathway. mRNA expression levels of (a) Nrf2, (b) NOQ-1, and (c) HO-1 in control siRNA and siRNA PPAR*β*/*δ* cells incubated in low- (LG) or high-glucose (HG) medium for 24 h, in the presence or absence of GW0742 (GW, 10^−6^ M). mRNA data presented as a ratio of arbitrary units of mRNA (2^−ΔΔCt^). All data are mean ± SEM (*n* = 8), and experiments were repeated at least three times independently. ^∗^*P* < 0.05 versus LG. ^#^*P* < 0.01 and ^##^*P* < 0.01 versus HG.

PPAR*β*/*δ*-induced downregulation of Nrf2 signaling was confirmed by siRNA-based downregulation of PPAR*β*/*δ*. HUVECs transfected with PPAR*β*/*δ*-specific siRNA showed a marked suppression of GW0742-induced upregulation of Nrf2 (Figure 4(a)) and NQO-1 (Figure 4(b)) under high-glucose condition, while the expression of HO-1 was abolished in both conditions (Figure 4(c)).

### 3.3. Effects of PPAR*β*/*δ* Agonists on Intracellular ROS Production

**Figure 5:**
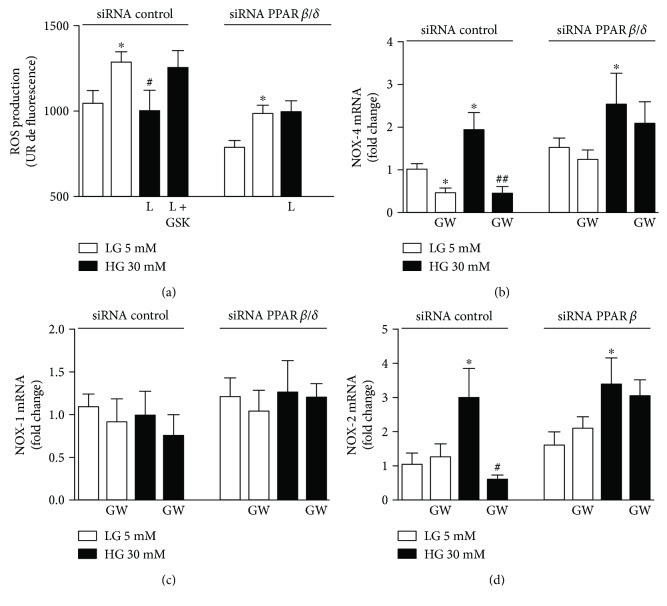
Effect of PPAR*β*/*δ* agonists in intracellular ROS production. (a) ROS and (b–d) mRNA expression levels of NOX-4 (b), NOX-1 (c), and NOX-2 (d) in HUVEC transfected with PPAR*β*/*δ*-specific siRNA (siRNA PPAR*β*/*δ*) incubated in low- (LG) or high-glucose (HG) medium for 24 h in the presence or absence of either L165041 (L, 10^−6^ M) or GW0742 (GW, 10^−6^ M), respectively. GSK0660 (10^−6^ M) was added 30 min before the incubation with L165041. mRNA data presented as a ratio of arbitrary units of mRNA (2^−ΔΔCt^). All data are mean ± SEM (*n* = 8), and experiments were repeated at least three times independently. ^∗^*P* < 0.05 versus LG. ^#^*P* < 0.01 and ^##^*P* < 0.01 versus HG.

Oxidative stress induced by hyperglycemia has been reported in endothelial cells [20]. Accordingly, we observed higher levels of ROS induced by 30 mM of glucose compared with baseline conditions (5 mM glucose) (Figure 5(a)). The PPAR*β*/*δ* agonist L165041 (10^−6^ M) abolished ROS production under the high-glucose condition, indicating its protective potential at the level of reactive species generation. Furthermore, coincubation of this PPAR*β*/*δ* agonist with GSK0660 or PPAR*β*/*δ*-specific siRNA suppressed its efficacy to inhibit high glucose-induced ROS overproduction (Figure 5(a)).

HUVECs, under high-glucose condition, showed markedly upregulated mRNA abundance of NOX-4 (Figure 5(b)) and NOX-2 (Figure 5(d)) while the expression of NOX-1 was not significantly affected (Figure 5(c)).

In experimental low-glucose condition, the coincubation of HUVECs with GW0742 downregulated the mRNA abundance of NOX-4 (Figure 5(b)); this NOX-4 downregulation seems to be linked with the capacity to elevate in both gene and protein expressions of HO-1 expression as a gene target of PPAR*β*/*δ*. Interestingly, the potential of GW0742 to inhibit the increase of mRNA abundance of NOX-4 and NOX-2 induced by high glucose was blunted by the siRNA-mediated downregulation of PPAR*β*/*δ* (Figures 5(b) and 5(d)).

### 3.4. Effect of GW0742 on Blood Glucose Levels of Control and Diabetic Rats

**Figure 6:**
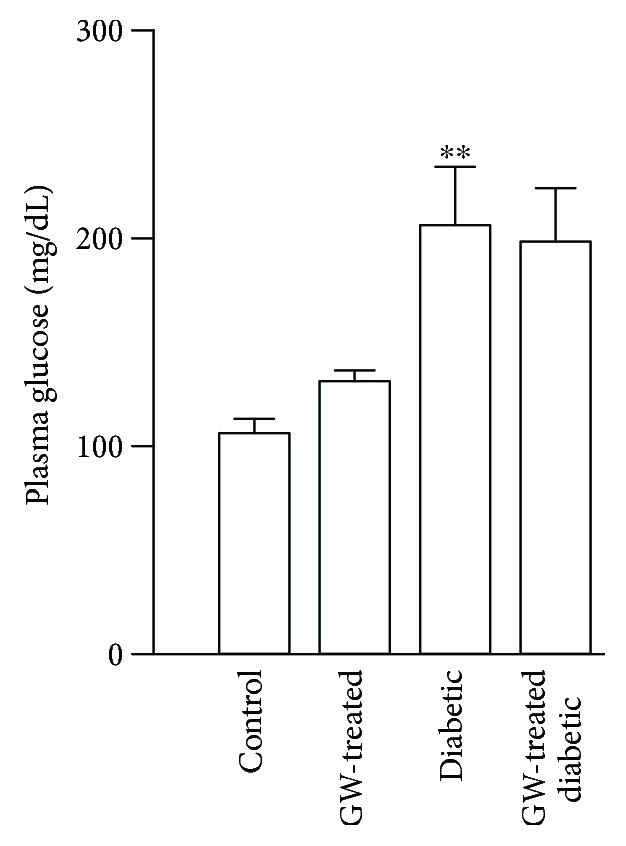
Effect of GW0742 on blood glucose levels of control and diabetic rats. Plasma glucose concentrations were measured by colorimetric method. Values are expressed as mean ± SEM of *n* = 8–10 rats. ^∗∗^*P* < 0.01, diabetic versus control rats.

STZ diabetic rats showed hyperglycemia evidenced by the significantly elevated levels of fasting blood glucose as compared to the control group. In both experimental groups, the long-term GW0742 administration did not alter the levels of glucose (Figure 6), indicating that the protective effect of PPAR*β*/*δ* agonist was glucose-independent.

### 3.5. Effects of Oral GW0742 on Nrf2 Pathway in Aorta of Diabetic Rats

**Figure 7:**
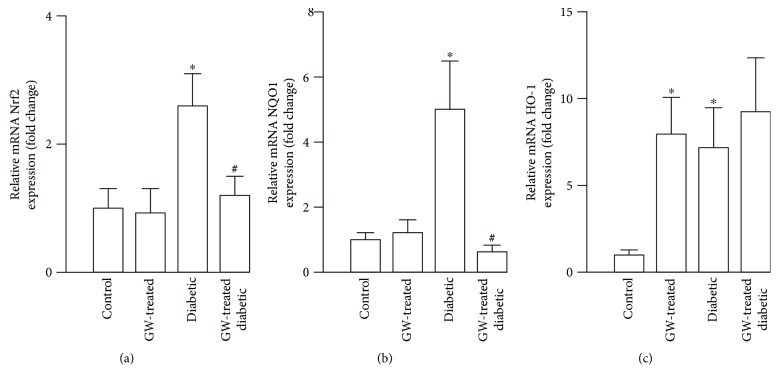
Effect of oral GW0742 on the Nrf2 pathway in the aorta of diabetic rats. mRNA expression of (a) Nrf2, (b) NQO-1, and (c) HO-1 in the aorta of all experimental groups. Data are presented as the ratio of arbitrary units of mRNA (2^−∆∆Ct^). Results are shown as mean ± SEM *n* = 8–10 rats. ^∗^*P* < 0.05, diabetic versus control group. ^#^*P* < 0.05, GW0472-treated diabetic versus nontreated diabetic rats.

Aortas from STZ diabetic rats showed a marked increase in Nrf2 (Figure 7(a)), NQO-1 (Figure 7(b)), and HO-1 (Figure 7(c)) mRNA expression as compared to the control rats. Chronic treatment with GW0742 downregulated Nrf2 and NQO-1 in the aortas of diabetic rats while it showed no effect on normal rat aorta. However, similar to the *in vitro* results, HO-1 mRNA abundance was higher in the vascular wall of control- and diabetic-treated rats (Figure 7(c)).

### 3.6. GW0742 Decreases Vascular ROS and NADPH Oxidase Activity

**Figure 8:**
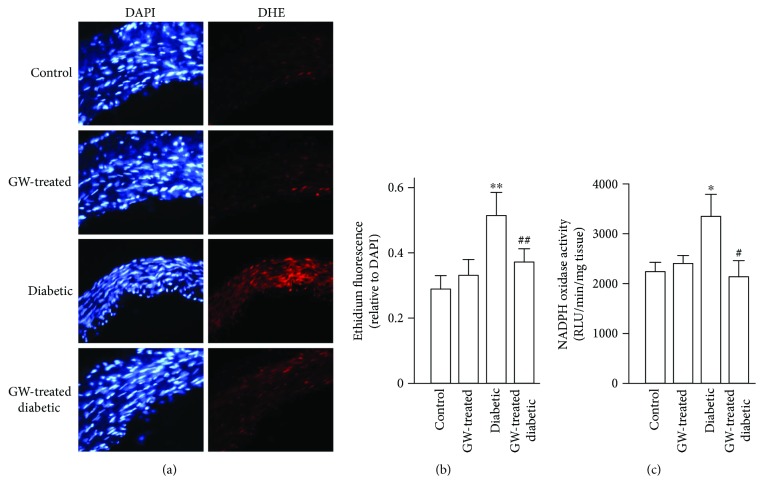
GW0742 decreases vascular ROS and NADPH oxidase activity. (a) The left panel shows blue fluorescence of the nuclear stain DAPI (×400 magnification), and the right panel shows arteries incubated in the presence of DHE which produces a red fluorescence when oxidized to ethidium by ROS. (b) Averaged values, mean ± SEM (*n* = 8–10 rings from different rats), of the red ethidium fluorescence normalized to the blue DAPI fluorescence. (c) NADPH oxidase activity measured by lucigenin-enhanced chemiluminescence (*n* = 6–10). ^∗^*P* < 0.05 and ^∗∗^*P* < 0.01, diabetic versus control group. ^#^*P* < 0.05 and ^##^*P* < 0.01, GW0472-treated diabetic versus nontreated diabetic rats.

To evaluate the effect of GW0742 on hyperglycemia-provoked oxidative stress, aortic rings from all experimental groups were stained with DHE and the activity of NADPH oxidase was determined. DHE staining revealed increased superoxide levels in the aorta of diabetic rats as depicted in Figures 8(a) and 8(b). In the same context, the aorta of diabetic rats showed significantly increased activity of NADPH oxidase (Figure 8(c)). While exerting no effect in normal rats, GW0742 suppressed ROS levels and NADPH oxidase in the aorta of STZ diabetic rats.

## 4. Discussion

Oxidative stress is a leading cause of ED and has previously been implicated in CV complications in diabetes [4]. Herein, we provide the first evidence that PPAR*β*/*δ* agonists can indirectly downregulate the Nrf2 pathway by suppressing HG-induced ROS accumulation in the vascular wall. Given the role of ROS in vascular diseases, our study suggests a potential therapeutic role of PPAR*β*/*δ* in diabetic vascular complications.

The role of hyperglycemia in diabetes vasculopathy has been well-acknowledged. Although the vascular endothelium has adaptive mechanisms to counteract hyperglycemia-induced oxidative stress, superfluous ROS levels induce endothelial dysfunction. In endothelial cells, increased superoxide generation represents the hallmark of hyperglycemia-mediated oxidative stress [23]. Accordingly, HUVECs exposed to HG and aorta of STZ diabetic rats showed markedly elevated levels of ROS. These findings are explained by the increased activity of NADPH oxidase which represents a major source of superoxide production under hyperglycemic conditions [17, 24]. Here, HUVECs showed increased NOX-4 expression following exposure to HG levels. NOX-4 is the prominent and major subunit of NADPH oxidase that provokes the generation of superoxide anions in the endothelium [25, 26]. Therefore, NOX-4 can induce oxidative damage [27] and endothelial injury [28] in response to cellular stress. Previous studies have also demonstrated increased NADPH oxidase in diabetic animals [9, 26] and human patients [29]. In addition, the increased levels of ROS could be attributed to hyperglycemia-induced eNOS uncoupling and mitochondrial electron transport chain as previously demonstrated [30–32]. Interestingly, a functional NOX-4 has been reported to be present in the mitochondria [33]. In a preparation of pure mitochondria, knockdown of NOX-4 by siRNA blocked mitochondrial superoxide generation induced by glucose [33]. Moreover, experimental evidence showed that genetic knockdown or inhibition of NOX-4 reduces ROS production in the vascular endothelium [27, 34]. Along with the upregulated NOX-4, HG-induced HUVECs showed increased expression of NOX-2, a NADPH oxidase subunit known to be expressed in a significant amount in HUVECs as well as in rat aortic endothelium [26].

GW0742 suppressed NADPH oxidase activity and NOX-4 induction and prevented oxidative stress, indicating that the antioxidative action of PPAR*β*/*δ* activation was related to its suppressive effect on NOX-4 activation induced by HG levels. Since both pharmacological inhibition and knockdown of PPAR*β*/*δ* abolished the suppressive effect of GW0742 on NOX expression and ROS generation, our results point to the specific inhibitory role of PPAR*β*/*δ* activation on HG-induced oxidative stress. These findings were confirmed *in vivo* where the treatment with GW0742 abolished superoxide generation and reduced NADPH oxidase in the aorta of STZ diabetic rats. Therefore, the PPAR*β*/*δ*-induced suppression of NOX-4 can significantly improve the integrity and prevent injury of the vasculature in diabetes. Accordingly, results from experimental animal models of STZ- and HFD-induced diabetes treated with GW0742 [12, 14] add support to our findings. Through its ability to activate PPAR*β*/*δ*, GW0742 significantly suppressed NADPH oxidase activity as well as the expression of its subunits, p47phox and p22phox, leading to reduced levels of superoxide in the diabetic aorta [12, 14].

The chronic administration of PPAR*β*/*δ* agonist did not reduce blood glucose levels in STZ diabetic rats, indicating that its beneficial vascular effects are independent of the glycemic state. Hence, we investigated the possible role of Nrf2/ARE/antioxidant signaling. In diabetes, activation of Nrf2 is an adaptive mechanism that protects the endothelium. This notion is being supported by several *in vitro* and *in vivo* studies. In bovine aortic endothelial cells, activation of Nrf2 pathway represented a defense mechanism against oxidative damage induced by advanced glycation end products [35] and hyperglycemia [32]. Consistent findings were elucidated by Ungvari et al. [20] in coronary endothelial cells where the adaptive induction of Nrf2 protected against the deleterious effects of hyperglycemia. In Nrf2(^+/+^) mice, HFD feeding elicited increased expression of HO-1 mRNA but not in Nrf2(^−/−^) mice [20]. In addition, HFD-fed Nrf2(^−/−^) mice showed increased vascular ROS levels and diminished vascular reactivity when compared with the Nrf2(^+/+^) mice received the same HFD, confirming the role of Nrf2 as an adaptive mechanism to counteract diabetes-associated endothelial dysfunction [20]. In our *in vitro* hyperglycemia model, HUVECs exhibited upregulated gene and protein expression of Nrf2 along with increased expression of NQO-1 and HO-1, adding support to the previous findings. Similarly, diabetic rats showed upregulated aortic Nrf2, NQO-1, and HO-1. In conjunction with the activated Nrf2 signaling, the aorta of the diabetic rats exhibited increased superoxide levels and NADPH oxidase activity. ROS produced by NOX activity can activate Nrf2 [36, 37] and hence NOX provides a feedback defense mechanism counteracting oxidative stress [38]. In the same context, Brewer et al. [39] demonstrated that NOX-4 has a potential role in regulating the redox status in cardiomyocytes *in vivo* via activating the Nrf2 pathway.

In our *in vitro* and *in vivo* models of hyperglycemia, PPAR*β*/*δ* activation significantly reduced Nrf2 and NQO-1 expression, whereas upregulating HO-1. These results corroborate the findings of Ali et al. [40], who explored the role of HO-1 in mediating the vasculoprotective efficacy of PPAR*β*/*δ* and its coactivator PGC1*α* against oxidant-induced injury. It can thus be suggested that PPAR*β*/*δ* agonists induce HO-1 independently of the Nrf2-pathway. HO-1 has been suggested as a protective factor against vascular oxidative stress and inflammation [41], and polymorphism of its gene promotor is associated with vascular diseases [41, 42]. Therefore, GW0742-induced upregulation of HO-1 observed herein contributed to the antioxidant potential of PPAR*β*/*δ*. In addition, the declined activity of NADPH oxidase via PPAR*β*/*δ* activation in our experimental models may be linked to the downregulation of Nrf2 and NQO-1. GW0742 has exerted a marked effect on the expression HO-1 as compared to L-165041. This is explained by the fact that GW0742 is a high-affinity PPAR*β*/*δ* agonist while L-165041 is a nonselective agonist.

In conclusion, this study shows, for the first time, that PPAR*β*/*δ* activation confers vascular protection against oxidative stress in diabetes via direct induction of HO-1 and downregulation of NOX-4 and, to a lesser extent, NOX-2. Additionally, the results show that, independent of the glycemic state, activation of PPAR*β*/*δ* diminished ROS levels, NADPH oxidase activity, and expression of Nrf2 and NQO-1. This research highlights the potential of PPAR*β*/*δ* agonists as novel therapies to reduce vascular complications of diabetes.

## Figures and Tables

**Table 1:** Oligonucleotides for real-time qRT-PCR.

mRNA targets	Descriptions	Species	Sense	Antisense
*NRF2*	Nuclear factor erythroid 2-related factor 2	*Homo sapiens*	GAGAGCCCAGTCTTCATTGC	AGTTTGGCTTCTGGACTTGG
*HO-1*	Heme oxygenase-1	*Homo sapiens*	AAGTATCCTTGTTGACACG	TGAGCCAGGAACAGAGTG
*NQO1*	NAD(P)H quinone dehydrogenase 1	*Homo sapiens*	AGACCTTGTGATATTCCAGTTC	GGCAGCGTAAGTGTAAGC
*NOX1*	Nox-1 subunit of NADPH oxidase	*Homo sapiens*	TCTTGCTGGTTGACACTTGC	TATGGGAGTGGGAATCTTGG
*NOX2*	Nox-2 subunit of NADPH oxidase	*Homo sapiens*	CCTAAGATAGCGGTTGATGG	GACTTGAGAATGGATGCGAA
*NOX4*	NOX-4 subunit of NADPH oxidase	*Homo sapiens*	AGTCAGCTCTCTCCTTTCAGG	CTTGCCCCCTTTGAATAAAT
*GAPDH*	Glyceraldehyde-3-phosphate dehydrogenase	*Homo sapiens*	AACGAATTTGGCTACAGC	AGGGTACTTTATTGATGGTACAT
*NRF2*	Nuclear factor erythroid 2-related factor 2	*Rattus norvegicus*	GTTGAGAGCTCAGTCTTCAC	CAGAGAGCTATCGAGTGACT
*HO-1*	Heme oxygenase-1	*Rattus norvegicus*	GCACAGGGTGACAGAAGAGG	ATGGCATAAATTCCCACTGC
*NQO1*	NAD(P)H dehydrogenase: quinone 1	*Rattus norvegicus*	GGGATATGAATCAGGGAGAGG	TGCCCTAAACCACAGAGAGG
*Actb*	*β*-Actin	*Rattus norvegicus*	AATCGTGCGTGACATCAAAG	ATGCCACAGGATTCCATACC
